# Loss of RAB25 Cooperates with Oncogenes in the Transformation of Human Mammary Epithelial Cells (HMECs) to Give Rise to Claudin-Low Tumors

**DOI:** 10.1155/2024/8544837

**Published:** 2024-05-20

**Authors:** Pooja Joshi, Vijayalakshmi Ayyagari, Samikshya Kandel, Vishnu Modur, Muhammad F. Iqbal, Kathy Robinson, John Gao, Krishna Rao

**Affiliations:** ^1^Arrakis Therapeutics, Waltham, MA, USA; ^2^Department of Obstetrics and Gynecology, Southern Illinois University School of Medicine, Springfield, IL 62702, USA; ^3^Department of Medical Microbiology, Southern Illinois University School of Medicine, Springfield, IL 62702, USA; ^4^Medpace, Inc., 5400 Medpace Way, Cincinnati, OH 45227, USA; ^5^Cancer Specialists of North Florida, 80 Pinnacles Drive, Suite 700, Palm Coast, FL 32164, USA; ^6^Simmons Cancer Institute at Southern Illinois University, 315 W Carpenter St., Springfield, IL 62702, USA; ^7^Department of Pathology and Gastroenterology, Memorial Medical Center, Springfield, IL, USA

## Abstract

The loss of RAB25 expression—RAS superfamily of GTPase characteristic of numerous breast cancers—corresponds with H-RAS point mutations, particularly in triple-negative breast cancers (TNBC), a subtype associated with a poor prognosis. To address the poorly understood factors dictating the progression of TNBC tumors, we examine the cooperative effects that loss of RAB25 expression in human mammary epithelial cell (HMEC) lines with H-RAS mutations confers in tumorigenesis. HMECs were immortalized by transduction with LXSN CDK4 R24C, a mutant form of cyclin-dependent kinase, followed by transduction with hTERT, a catalytic subunit of the telomerase enzyme. We found that with the loss of RAB25 and overexpression of mutant H-RAS61L, immortal HMECs transformed toward anchorage-independent growth and acquired an increased ability to migrate. Furthermore, cells express low CD24, high CD44, and low claudin levels, indicating stem-like properties upon transformation. Besides, loss of RAB25 and overexpression of H-RAS61L resulted in increased expression of transcription factors Snail and Slug that drive these cells to lose E-cadherin and undergo epithelial-mesenchymal transition (EMT). This study confirms that loss of RAB25 and overexpression of mutant H-RAS can drive HMECs toward a mesenchymal stem-like state. Our findings reveal that RAB25 functions as a tumor suppressor gene, and loss of RAB25 could serve as a novel biomarker of the claudin-low type of TNBC.

## 1. Introduction

Breast cancer is the most prevalent cancer type in American women; nearly one in eight women develop breast cancer. Several oncogenes and tumor suppressor genes such as RAS, BRCA-1, BRCA-2, and BCL-2 contribute to tumorigenesis of the mammary gland [1]. The genomic characterization of human breast tumors done by Hu et al., Herschkowitz et al., and Parker et al. identified six biologically distinct subtypes—luminal A, luminal B, basal-like, HER2-enriched, claudin-low, and normal-like. In the United States, around 15-20% of all breast cancers are triple-negative breast cancer (TNBC), and women under the age of 40 are at a higher risk of developing TNBC [2]. Up to 19% of women diagnosed with TNBC carry BRCA1 and BRCA2 germline mutations [3, 4]. By gene expression analysis, the TNBC were classified into six subtypes: basal-like (BL-1 and BL-2), immunomodulatory (IM), mesenchymal-like (M), mesenchymal stem-like (MSL), and androgen receptor type (AR) [5], each subtype with different molecular and clinical characteristics.

Nearly 30% of all human cancers carry RAS mutations. Different RAS isoforms (H-RAS, K-RAS, and N-RAS) are mutated in different cancer types, activate distinct pathways, and differ in their transforming ability [6]. Expression or activation of H-RAS, often associated with breast cancer, is considered a marker for aggressiveness in breast tumors (tumor) [7, 8]. The RAB (Ras-related in the brain) proteins belonging to the RAS superfamily of RAS GTPases regulate the vesicular trafficking machinery. More than 70 different RAB proteins have been identified in the human genome, reflecting the complexities of transport machinery inside the cell. As a measure of this complexity, distinct RAB proteins are expressed in specific cell types that carry out a particular function. The vesicular trafficking machinery by RAB GTPases delivers vesicles of intracellular and extracellular cargos, including growth factors [9], nutrients, integrins [10, 11], and other junction proteins, that control cellular signaling that controls growth, development [12], locomotion, and overall survival [13]. Elevated levels of RAB25 are reported in the ER and PR positive subtypes of breast cancer [14] and in the ER and PR negative breast cancer subtypes; we see the loss of RAB25 leading to activation of the RAS signaling pathway [15, 16]. Loss of RAB25 is seen in TNBC and acts via multiple pathways to suppress apoptosis and promote angiogenesis by altering VEGF expression [17].

Studies have reported that the dysregulation of RAB25 gene expression could be associated with the aggressiveness of various human cancers, including breast and ovarian cancers [18, 19], prostate cancer [20], bladder carcinoma [21], lung cancer [22], bladder cancer [21], gastric carcinoma [23], head and neck cancer [24, 25], and esophageal carcinoma [26].

We have previously shown the importance of loss of RAB25 expression in breast cancer [15, 16], and RAB25 serves as a tumor suppressor in TNBC [17]. We observed RAB25 loss in transformed immortalized human mammary epithelial cell lines like RAO-3 and RAO-4 that were associated with H-RAS oncogene mutations. We were interested in understanding if RAB25 loss is associated explicitly with H-RAS mutations or even other oncogenes commonly mutated in breast cancer. To test this, we chose the following oncogenes: H-RASV12G, H-RAS61L, and IGF1-R; around 50% of breast tumors express activated IGF1-R [27, 28].

Our results show that the loss of Rab25 coupled with the overexpression of mutant H-RAS61L transforms HMEC and drives cells to undergo epithelial to mesenchymal transition, as evidenced by the acquisition of stem-like properties giving rise to mesenchymal stem-like triple-negative breast tumors.

## 2. Materials and Methods

We followed methods previously described by Joshi et al. [29], and those sections are quoted.

### 2.1. Isolation and Culture of Human Mammary Epithelial Cells

“The human mammary epithelial cells (HMEC 5.6) were derived from an individual undergoing reduction mammoplasty with no known breast pathology, as confirmed by the histopathology of the postsurgical specimens. Specimens were obtained from the Simmons Cancer Institute tissue bank under the IRB-approved protocol # 08-112/12-177. HMECs were prepared by the method of Smith et al. The primary HMECs were derived from mammary tissues that were obtained from mammoplasty. Mammary tissue was then cut into pieces and digested in a mixture of 14 U/ml hyaluronidase, 15 U/ml collagenase, 10 *μ*g/ml insulin, 1x pen/strep, and 10% FCS in DFCI-1 medium at 37°C overnight. Cells were sieved through a sterile strainer, washed in PBS, spun down at 1500 rpm for 5 minutes, and plated on a 10 cm plate. Fibroblasts were removed by trypsin/EDTA treatment. HMEC were immortalized by transducing with a zeocin-based vector carrying hTERT and a neomycin-based construct that contained a mutant CDK4 (R24C). The antibiotic concentrations for selection are as follows: G418: 100 *μ*g/ml, zeocin: 20 *μ*g/ml, puromycin: 2.5 *μ*g/ml, and Hygromycin: 20 *μ*g/ml. The cell line is sensitive to Puromycin and Hygromycin. HMEC was grown at 37°C and 5% CO_2_ in DFCI-1 media supplemented with 10% fetal bovine serum (FBS), 100 units per ml penicillin, and 100 *μ*g/ml streptomycin according to the standard protocol. The cells have been passaged a minimum of 20 times to ensure immortality. RAO-1 is a human mammary epithelial cell (HMEC) line obtained from healthy individuals undergoing reduction mammoplasty and was immortalized by transduction with the catalytic subunit of telomerase (hTERT) after passage through stasis. RAO-4 is derived from RAO-1 by transduction with the Q61L mutant H-RAS gene, and RAO-4 forms human mammary epithelial carcinoma when injected into nude mice [15, 30].”

### 2.2. Construction of Plasmid

To generate Rab25 knockdown stable cell lines, RAB25 siRNA and control were purchased (ABM, Richmond, Canada). Rab25 and control siRNAs were transfected into a growing culture of 293 T cells with the aid of packing vectors and Lipofectamine transfection reagent (Invitrogen, NY, USA). The resulting viral supernatant after 48 hours was collected and introduced to a growing culture of HMEC 5.6 and HMEC 2.6 with the aid of polybrene reagent (Millipore, MA, USA). Stable HMEC 5.6-KD and HMEC 2.6-KD (knockdown) clones were obtained by Puromycin selection in vitro. To generate a Hygromycin-based retroviral vector carrying oncogenes, pCGN H-RAS61L (Plasmid #14720, Addgene), pWZL Hygro H-RAS V12 (Plasmid #18749, Addgene), pBABE-bleo IGF-1R (Plasmid #11212, Addgene), pWZL Hygro (Plasmid #18750, Addgene), and pLENTI-EF1a-GFP-2A-Hygro (Plasmid #LV067, ABM) vectors were purchased. Plasmid DNA was extracted by using the QIA Spin Miniprep Kit following the manual instructions. The vectors were subjected to restriction digestion with the enzymes flanking the gene of insert and site of insertion in MCS, respectively. Upon gel extraction of the insert oncogenes, they were cloned into the control pWZL Hygro vector. Bacterial colonies were subjected to ampicillin selection at 100 *μ*g/ml (Thermo Fisher Scientific, MA, USA). Restriction digestion was done to confirm the bacterial-positive colony carrying the gene of interest. This was followed by the extraction of the plasmid DNA using the QIAGEN Plasmid Midi Kit, following the manufacturer's instructions. The plasmid DNA was stored at -20°C for future use.

### 2.3. Oncogene Overexpression and Selection

Mammalian expression vectors with different oncogene inserts and control vectors were transfected into Phoenix cells cultured in DMEM media, and Lipofectamine was used as the transfection agent (Invitrogen, NY, USA) along with viral packaging vectors. The viral supernatant was harvested after 48 hours and used to infect the growing cultures of HMEC 5.6 RAB25- and HMEC 2.6 RAB25- cells by using a polybrene reagent (Millipore, MA, USA). The resulting viral supernatant after 48 hours was collected and introduced to a growing culture of HMEC 5.6 RAB25- cells with the aid of polybrene reagent (Millipore, MA, USA). The transfected cells were subjected to Hygromycin selection (20 *μ*g/ml) in vitro (Gold Biotechnology, MO, USA) over the next few weeks. The surviving clones were harvested separately by using sterile Cloning Cylinders (Fisher Scientific, MA, USA) and seeded in separate tissue culture plates.

### 2.4. mRNA Extraction, cDNA Synthesis, and RT-PCR

“RNA was isolated from cells using the QIAGEN Kit. The cDNA was prepared by reverse transcription using the RevertAid First Strand cDNA synthesis kit (Thermo Scientific) and used as a template for RT-PCR (GoTaq qPCR Promega kit). The RT-PCR reaction was run on an ABI cycler using the primer sequences listed below. The relative mRNA expression level was determined by the comparative CT method and normalized to relative *β*-actin expression.” Error bars represent the standard deviation of the mean. The primers used are given in Table 1.

### 2.5. Western Blot

“Cells were washed with PBS and incubated with RIPA (Cell Signaling Technology) lysis buffer on ice. The collected lysate was spun down, and a Bradford assay was used to determine the protein concentration in the samples. Around twenty micrograms of protein from each sample was run through a 12% polyacrylamide gel. Proteins were transferred to a nitrocellulose blotting membrane (Amersham Protan 0.2 *μ*m, GE Healthcare Life Sciences) and were blocked for an hour at room temperature by using TBS (10 mM Tris-HCL, 150 mM NaCl, 0.1% Triton X-100, and pH 8.0) buffer containing 5% skim milk. The membranes were incubated with the following antibodies overnight on a shaker at 4°C. The membranes were then washed three times for five minutes each with TBST (TBS buffer with 0.05% Tween 20). The membranes were incubated with respective secondary antibodies for one hour at room temperature on a shaker.” Primary antibodies used were Mouse monoclonal *β* actin (Cell Signaling Technology, MA, USA), Rabbit polyclonal RAB25 (Abcam), Mouse monoclonal H-RAS (Thermo Fisher Scientific, MA, USA), Rabbit monoclonal E-cadherin (Cell Signaling Technology, MA, USA), Rabbit monoclonal claudin (Cell Signaling Technology, MA, USA), Goat polyclonal SNAIL (Santa Cruz Biotechnology, TX, USA), and Rabbit monoclonal IGF-1 Receptor (Cell Signaling Technology, MA, USA).

### 2.6. Flow Cytometry

To detect the expression pattern of stem cell markers CD24, CD44, and ALDH1 in RAO-1, RAO-2, RAO-4, and HMEC with loss of Rab25 and mutant H-Ras61L, flow cytometry was performed following the protocols from the BD Cytofix/Cytoperm™ Fixation/Permeabilization Solution Kit. Briefly, cells were harvested by dissociation using 0.25% trypsin with 0.02% EDTA and suspended in ice-cold FACS buffer containing PBS and 0.5% BSA. Further, cells were fixed in a fix/perm solution following the manufacturer's instructions and stained using primary conjugated CD24 Mouse anti-Human-FITC, CD44 Mouse anti-Human-APC, and Anti-ALDH1A1 Antibody (PE), Mouse Monoclonal for 30 minutes at room temperature. Separate staining with Invitrogen™ Propidium Iodide Ready Flow™ Reagent as a viability control and unstained cells were also included in the experiment. After washing twice with FACS wash buffer, analysis was performed using Accuri C6 Plus.

### 2.7. Migration Assay

“Cell culture inserts were placed in 24 well plates, and 100 *μ*l of serum-free media was added to the chamber along with 200 *μ*l of cells resuspended in serum-free media after trypsinization. Around 700 *μ*l of culture media with serum was added to the lower chambers and allowed to incubate at 37°C for 12-16 hours. After incubation, the media was removed, and the chambers were washed twice with PBS. The cells were fixed by using formaldehyde (3.7% in PBS) for 2 minutes at room temperature. The cells were made permeable by adding 100% methanol. Cells were counted by staining them with Giemsa for 15 minutes at room temperature.”

### 2.8. Colony Forming Assay

Cells in culture were harvested with trypsin-EDTA and resuspended in DFCI media, and around 200 cells were seeded in each well of a six-well plate. The formation of colonies was assayed 12 to 14 days later after plating of the cells. Cells were fixed by using 4% paraformaldehyde for 5 minutes at room temperature. Cells were stained by using Crystal Violet for 15 minutes at room temperature. The number of colonies formed was counted manually, and images were taken. Experiments were repeated in triplicate.

### 2.9. Wound Healing Assay

Around 6000 cells were seeded in each well of a six-well plate, and cells were allowed to reach a monolayer confluency. A clean, sterile 200 *μ*l pipette tip was used to make a scratch across the well. Cells were monitored over a period of 24 hours till the cell gap was closed. Images were taken every 6 hours from time zero (wound created) till the wound closure. The distance migrated by the cells was calculated.

### 2.10. Mammosphere Forming Assay

Culture cells were harvested in trypsin-EDTA and carefully resuspended in DFCI media supplemented with 1% N2 supplement, 2% B27 supplement, 20 ng/ml b-FGF-2, and 20 ng/ml EGF (GIBCO Life Technologies, NY, USA). Around 5000 cells were counted and plated in each well of a six-well ultralow-attachment plate (Corning, Lowell, MA). Mammospheres were assayed 10 to 14 days after cell plating. Mammospheres > 40 *μ*m were counted manually under a light microscope, and experiments were done in triplicate. The primary mammospheres formed were resuspended in the media to obtain a single-cell suspension. Cells were trypsinized and seeded in fresh supplemented DFCI media in a six-well ultralow-attachment plate to assess for secondary mammosphere formation. Mammospheres > 40 *μ*m in size were counted manually.

### 2.11. Tumor Samples

Mammary spindle cell carcinoma specimens from 13 patients were obtained after the Institutional Review Board (IRB), i.e., the Springfield Committee for Research Involving Human Subjects (SCRIHS) approval. The tissue samples were obtained through the SIU Cancer Institute Tumor Bank and Memorial Medical Center. The paraffin-embedded tissue samples were verified and deidentified before the process.

### 2.12. Immunohistochemistry

Immunohistochemical studies were performed on paraffin-embedded tissue sections. Deparaffinization (immunohistochemistry protocol for paraffin-embedded tissue sections, Cell Signaling Technology, MA, USA) was initially carried out, followed by the blocking of nonspecific antigen binding sites by using a universal blocking buffer for one hour at room temperature. Tissue sections were incubated with primary antibodies Rabbit polyclonal RAB25 (Abcam) and Mouse monoclonal Snail (Cell Signaling Technology, MA, USA) for one hour at room temperature, followed by incubation with the respective HRP-tagged secondary antibody for one hour at room temperature. The sections were washed two times for five minutes each with PBST. Immunostaining was detected by using the diaminobenzidine (DAB substrate) method (Cell Signaling Technology, MA, USA) according to the manufacturer's protocol. The peroxidase activity was detected in a DAB working solution containing DAB chromogen concentrate and DAB diluent. The tissue sections were counterstained with Mayer's hematoxylin. The slides were washed with double-distilled water and PBS and mounted with coverslips by using Histomount (National Diagnostics, GA, USA) upon complete drying. The samples were assessed for positive and negative staining by imaging under bright-light microscopy.

### 2.13. Western Blot Analysis for RAB25 and HIF-1 Alpha in RAO Series Cell Lines

For Rab25, cell lysates were prepared by lysing the cells in a buffer containing 10 mM Tris-HCL, pH 7.4, 100 mM NaCl, 1 mM EDTA, 1 mM EGTA, 1% Triton X-100 0.5% NP-40, 1 mM PMSF, 50 *μ*g/ml aprotinin, and 10 *μ*g/ml leupeptin and incubating on ice for 30 min. A total of 50 *μ*g of protein from each sample was loaded onto a 12% SDS polyacrylamide gel and subjected to electrophoresis and western blotting. The membrane was incubated with anti-Rab25 monoclonal mouse IgG (ProMab, Albany, CA) overnight at 4°C. The membrane was further incubated with secondary antibodies conjugated to IRDye (Li-COR) and analyzed using OdysseyH imaging software 3.0. Antibody to tubulin (monoclonal mouse IgG, Oncogene, San Diego, CA) was used as a control for protein loading.

For HIF-1 alpha detection, cells grown to 80% confluence were treated with 100 *μ*M CoCl_2_ in DFCI-media for 20 hours. Following incubation, cells were lysed in lysis buffer containing 10 mM Tris-HCL, pH 7.4, 100 mM NaCl, 1 mM EDTA, 1 mM EGTA, 1% Triton X-100 0.5% NP-40, 1 mM PMSF, 1X protease inhibitor cocktail (Sigma), 1 mM DTT, and 1 mM sodium vanadate. Lysate was heat-inactivated at 95°C for 5 min. A total of 100 *μ*g of protein was loaded onto a 10% SDS-PAGE gel and electrophoresed. Following the overnight transfer of proteins to the immobilon P membrane at 30 V (constant), the membrane was blocked with OdysseyH blocking buffer and incubated with primary antibody overnight at 4°C (1 : 200 dilution in block buffer, Novus Biologicals). The membrane was further incubated with secondary antibodies and analyzed as described above.

For GLS2 detection, cell lysates of RAO-1-4 were prepared by lysing the cells in a 1x RIPA buffer containing Triton X-100 0.5% NP-40, 1 mM PMSF, 50 *μ*g/ml aprotinin, and 10 *μ*g/ml leupeptin. A total of 25 *μ*g of protein from each sample was loaded onto a 12% SDS polyacrylamide gel and subjected to electrophoresis and western blotting. The membrane was incubated overnight at 4°C with anti-GLS2 monoclonal mouse IgG (Novus Biologicals, Littleton, CO) and detected as described above. Antibody to *β* actin (monoclonal mouse IgG, Oncogene, San Diego, CA) was used as a control for protein loading.

### 2.14. Cytogenetics

Metaphase chromosomes from HMEC 5.6 RAB25 were prepared by using standard cytogenetic techniques. The cells were incubated with 0.06 *μ*g/*μ*l of colcemid at 37°C for 2.5 hours and trypsinized with trypsin/EDTA (0.5%/0.1%). The cells were then incubated in 2 ml of 0.075 M potassium chloride for thirty minutes at 37°C. This was followed by the addition of 1 ml of Carnoy's fixative (3 : 1 by volume, methanol : acetic acid) and incubation at room temperature for ten minutes. The fixed cells were then spun down and resuspended in 2 ml of Carnoy's fixative. The prepared metaphase chromosomes were sent to Cell Guidance Systems (St. Louis, MO, USA) for G-banding (Wright's staining) and analysis. Twenty cells were analyzed after G-banding, and the karyotypes were described as per the International System for Human Cytogenetic Nomenclature (ICSN).

### 2.15. RAS Mutational Studies

Paraffin-embedded tissue sections were sent to Neogenomics (Fort Myers, FL) to detect H-RAS and K-RAS mutations in three of our 100% spindle cell carcinoma cases. DNA was isolated from microdissection-enriched FFPE tissue. Both exons of the H-RAS gene, 1 and 2, are analyzed by bidirectional Sanger sequencing. Mutations were evaluated for entire K-RAS exons 2 and 3 by high-sensitivity Sanger sequencing. This includes codons 12, 13, 59, and 61.

### 2.16. Xenograft Assay

“Five-week-old female nu/nu mice were exposed to gamma-irradiation (300 rads) to suppress NK cell activity and tumor intake. Tumor cells were trypsinized and washed twice with PBS. 5 × 10^6^ cells were injected orthotopically into the mammary fat pad. Tumor volume was monitored twice a week by using calipers. The formula to calculate tumor volume was as follows: (length × width^2^)/2.”

### 2.17. Clinical Data

We collected data from patients diagnosed with spindle cell breast cancer between January 1, 2001, and January 31, 2015, at Southern Illinois University School of Medicine and affiliated hospitals, by retrospective medical record review. Study approval was obtained from the IRB.

Patients diagnosed with spindle cell breast cancer and treated at Simmons Cancer Institute were identified using a database based on the ICD-9 codes. Inclusion criteria were (1) patients with spindle cell breast cancer and (2) the availability of electronic pathology reports, pathology slides, and therapy records. Exclusion criteria consisted of unavailable electronic medical records.

Data collection included demographic variables, clinicopathological and immunophenotypic features, treatments, and overall survival. Age at the time of diagnosis, gender, ethnicity, tumor staging, tumor size, histologic grade, pathologic features, extracapsular extension, lymphovascular invasion, details of surgery, chemotherapy, and overall survival were recorded.

The age at the time of diagnosis was recorded in years. Ethnicity was classified into Caucasians, African Americans, and others. The American Joint Committee on Cancer's sixth and seventh editions (corresponding to the year of diagnosis) of the AJCC Cancer Staging Manual were used for tumor staging. Pathology reports were used to determine the T and N classes, tumor grade, estrogen receptor (ER), progesterone receptor [31], and HER2-neu receptor status. Overall survival was the primary outcome measure, defined as the survival calculated (in months) from the date of diagnosis to death from any cause, and patients were censored at the last date known to be alive or Dec 31, 2016, whichever came first.

### 2.18. Statistical Analysis

Statistical analysis was performed using Student's *t*-test (two-tailed) in quantitative PCR experiments when two groups were compared. The values were expressed as the mean ± standard deviation. Data of in vitro assays were obtained from three repeats of independent experiments, and statistical analysis was performed using Student's *t*-test (two-tailed). Data with *P* < 0.05 was considered statistically significant, and *P* values > 0.05 and < 0.1 were considered to represent a trend. Fisher's exact *t*-test was used to determine the *P* value for IHC tumor sections.

## 3. Results

Our previous results have confirmed RAB25 as a tumor suppressor in breast cancer. However, it was unclear if its loss exclusively cooperated with H-RAS61L or perhaps with other oncogenes to promote transformation. To this end, immortal HMEC lines with a loss of RAB25 were created to observe the effects of RAB25 loss in vitro.

### 3.1. Reconfirmation of RAB25 Downregulation in HMEC Lines

**Figure 1:**
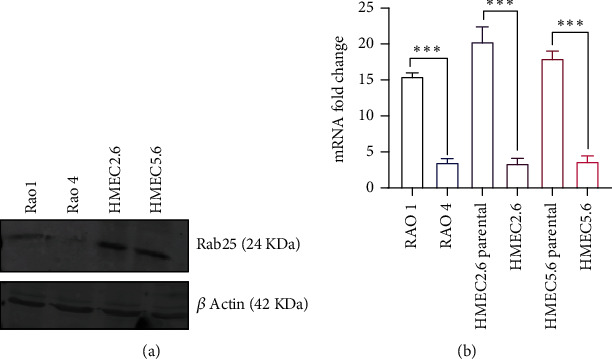
Reconfirmation of RAB25 downregulation in HMEC lines. (a) Rab25 knockdown in HMEC 5.6 and 2.6 cell lines at the protein level by siRNA technique. RAO-1 serves as a positive control. (b) Rab25 knockdown in HMEC 5.6 and 2.6 cell lines at the mRNA level. RAO-1 serves as a positive control. Any starred value indicates a *p* value of less than 0.05.

To investigate the role of oncogenes and loss of RAB25, we first reconfirmed shRNA-mediated RAB25 knockdown in human mammary epithelial cells 2.6 and 5.6 (HMEC). Quantitative PCR and western blots confirmed the expression levels of RAB25 at the mRNA and protein levels, respectively. RAB25 was successfully downregulated in HMEC 5.6 and 2.6 cell lines compared to the parental HMEC 5.6 and 2.6 cell lines (Figures 1(a) and 1(b)). These RAB25-downregulated cell lines became the model for our study.

### 3.2. Generation of Stable Cell Line Overexpressing H-Ras61L in Human Mammary Epithelial Cell Line 5.6 RAB25-

**Figure 2:**
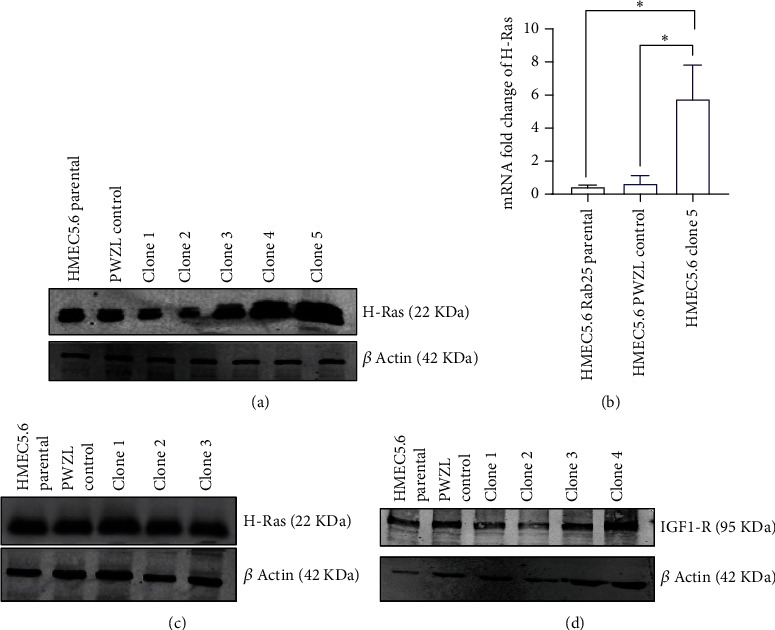
Overexpression of mutant H-Ras61L in HMEC 5.6 Rab25- cells. (a) Clone#5 shows the highest level of overexpression of H-Ras61L at the protein level. (b) Clone#5 shows a 5-fold increase in mRNA level expression of H-Ras compared to control and parental HMEC lines. (c) Overexpression of H-RASV12G (Clone#1). (d) Overexpression of IGF1-R (Clone#4) in HMEC 5.6 Rab25- at the protein level. Any starred value indicates a *p* value of less than 0.05.

We generated stable clones of the HMEC 5.6 RAB25- cell line overexpressing H-RAS61L to test our hypothesis following plasmid construction. Overexpression of H-RAS61L was confirmed at the mRNA level and protein level by qPCR and western blot, respectively (Figures 2(a) and 2(b)). Clone#5 appeared promising with the maximal overexpression of the oncogene, and thus a suitable model to confirm transformation using in vitro assays.

Additionally, we expressed other oncogenes, namely, H-RASV12G and IGF1-R in HMEC 5.6 RAB25- cells, and checked the expression level of these oncogenes at the protein level (Figures 2(c) and 2(d)). These oncogenes showed a significant increase in overexpression but did not result in transformation.

### 3.3. Loss of RAB25 Cooperates with Oncogene and Results in Increased Migration Ability and Increased Colony Formation

**Figure 3:**
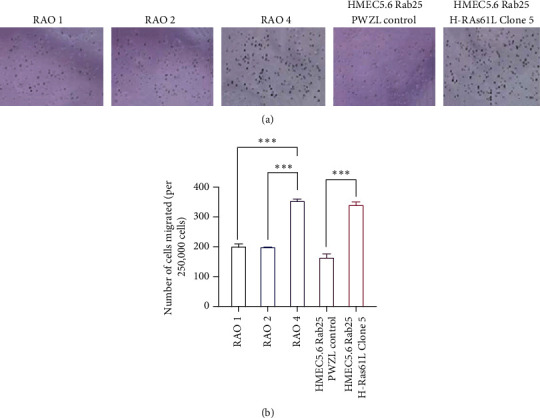
Loss of RAB25 cooperates with oncogene and results in increased migration. (a) Transwell migration assay showing increased migration in HMEC 5.6 Rab25- Clone#5 at the end of 16 hours and RAO-4 serves as a positive control. (b) Graphical representation of the number of cells migrated per 250,000 cells with standard error bars (*n* = 3). Any starred value indicates a *p* value of less than 0.05.

Despite substantial overexpression, as noted in Figure 2, the only oncogene to confer transformative properties with the loss of RAB25 was H-RAS61L. The invasive nature of HMEC 5.6 RAB25- Clone#5 cells was confirmed by a transwell migration assay, which showed increased migration compared to HMEC 5.6 RAB25- cells with the control vector (Figures 3(a) and 3(b)). These results are consistent with the migration ability of the transformed positive control cell line, RAO-4, and together confirm one of the properties seen in cells that have transformed. RAO-2 cells that express RAB25 and overexpress H-RAS61L did not show any increased migration ability, indicating that both RAB25 loss and overexpression of mutant RAS are necessary for cells to transform. Since HMEC 5.6 RAB25- Clone#5 cells showed a significant change in migration ability, we decided to carry out other further experimental studies on this clone alone.

**Figure 4:**
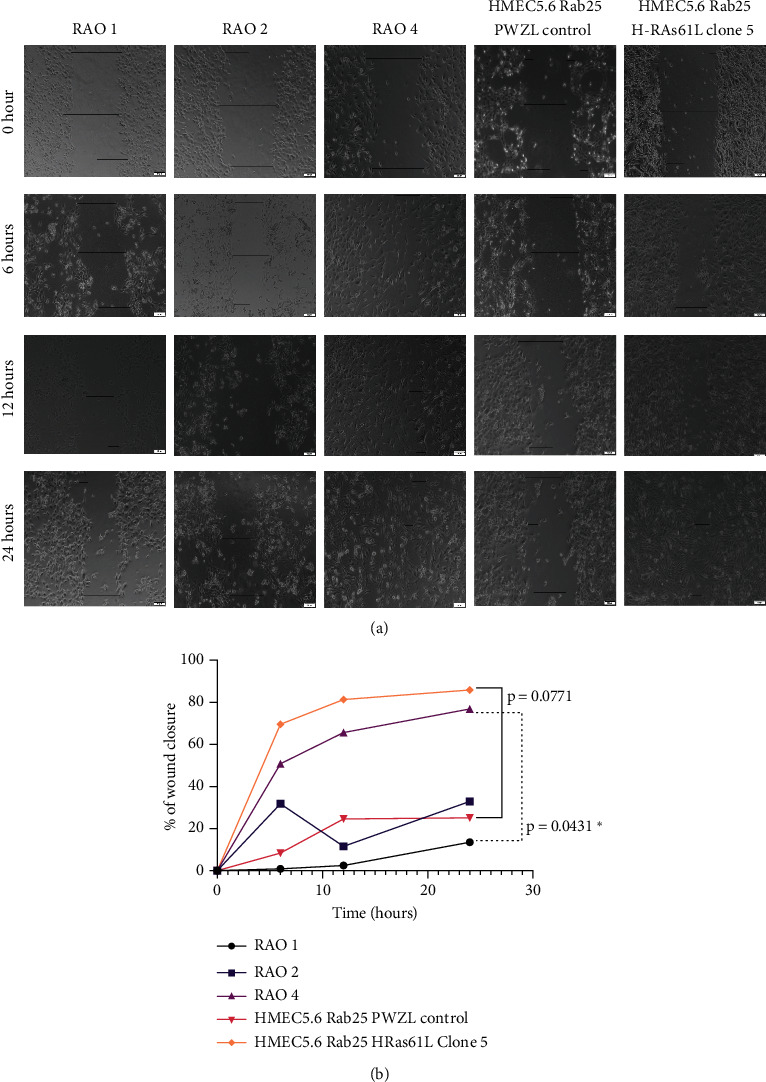
Loss of RAB25 cooperates with oncogene and results in increased invasion. (a) Wound healing assay was performed for RAO-1, RAO-2, RAO-4, HMEC 5.6 Rab25- pWZL control, and HMEC 5.6 Rab25- Clone#5. At the end of 24 hours, HMEC 5.6 Rab25- Clone#5 shows the highest level of cell invasion and RAO-4 served as the positive control. (b) Graphical representation of the distance covered by the cells with standard error bars (*n* = 3). Any starred value indicates a *p* value of less than 0.05.

**Figure 5:**
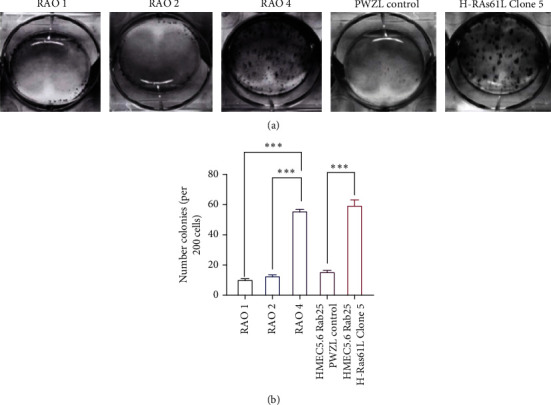
Loss of RAB25 cooperates with oncogene and results in an increase in colony formation. (a) Colony forming assay showing the formation of colonies in HMEC 5.6 Rab25- Clone#5 at the end of 14 days and RAO-4 serves as a positive control. (b) Graphical representation of the number of colonies formed per 200 cells with standard error bars (*n* = 3). Any starred value indicates a *p* value of less than 0.05.

The invasive nature of cells was tested by a wound healing assay. HMEC 5.6 RAB25- Clone#5 cells show 70% wound closure at the end of 24 hours, confirming the increased capacity of the cells to migrate. Similarly, the positive control cell line, RAO-4, had high migration properties by showing approximately 75% wound closure at the end of 24 hours. The negative control cell line RAO-1 cells and HMEC 5.6 RAB25- with pWZL control vector did not acquire increased migration; they only showed around 30% wound closure in 24 hours (Figures 4(a) and 4(b)). The migration results obtained from the wound healing assay are consistent with the transwell migration assay results. Anchorage-independent growth tested by the soft agar assay noted in Figure 5 shows that RAO-4 and HMEC 5.6 Clone#5 both displayed active colony formation in soft agar, confirming the anchorage-independent growth properties of both clones.

### 3.4. Overexpression of Mutant H-Ras61L Enhances Stem-Like Properties and Affects Expression of Stem Cell Markers

**Figure 6:**
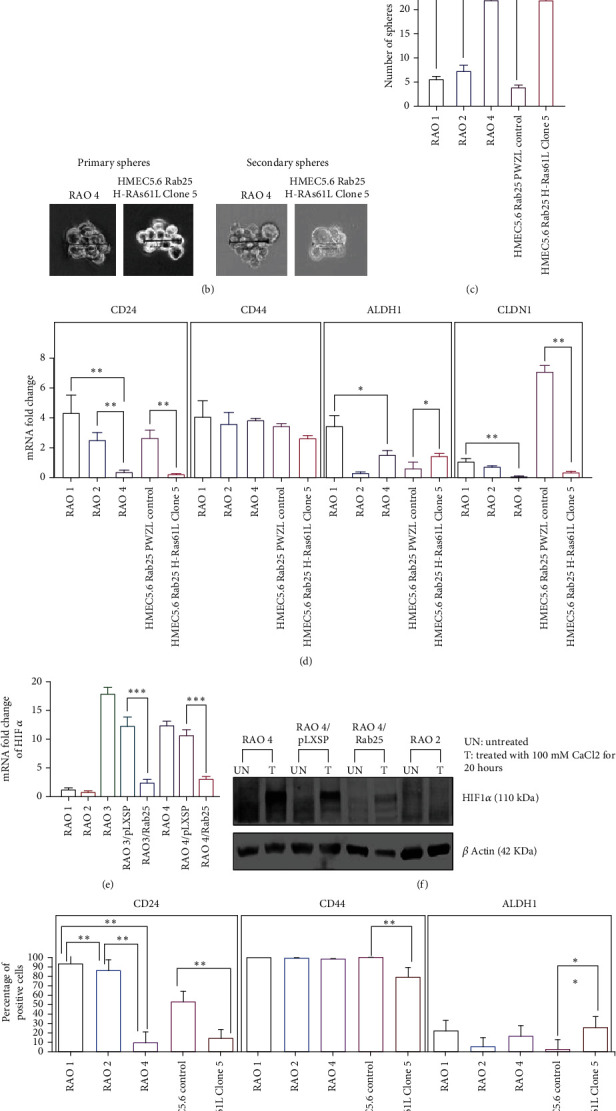
Loss of RAB25 and overexpression of mutant H-Ras61L enhances the stem-like property and affects the expression of stem cell markers. (a) Formation of mammospheres in HMEC 5.6 Rab25- Clone#5 and positive control RAO-4 after 10-14 days. (b) Formation of primary and secondary mammospheres with size > 40 *μ*m in HMEC 5.6 Rab25- Clone#5 and positive control RAO-4. (c) Graphical representation of the number of mammospheres formed with standard error bars (*n* = 3). (d) Graph showing the changes in the mRNA level expression of stem cell markers like CD24, CD44, ALDH1, and Claudin1 with standard error bars (*n* = 3). (e) HIF-1*α* mRNA levels are quite low in RAO-1 and RAO-2 but rise quite significantly in RAO-3 and RAO-4. RAB25 expression brings down HIF-1*α* levels quite significantly in RAO-3 and RAO-4. (f) Protein levels of HIF-1*α* as determined by western blotting mirrored real-time PCR results with a significant decline resulting from Rab25 expression. (g) Graph showing flow cytometry analysis of stem cell markers like CD24, CD44, and ALDH1. CD24 levels drop precipitously in both RAO-4 and Clone#5, while ALDH1 rises in both RAO-4 and Clone#5 (significantly). CD44 is stable in the RAO cell line series but does slightly drop in Clone#5. Any starred value indicates a *p* value of less than 0.05.

**Figure 7:**
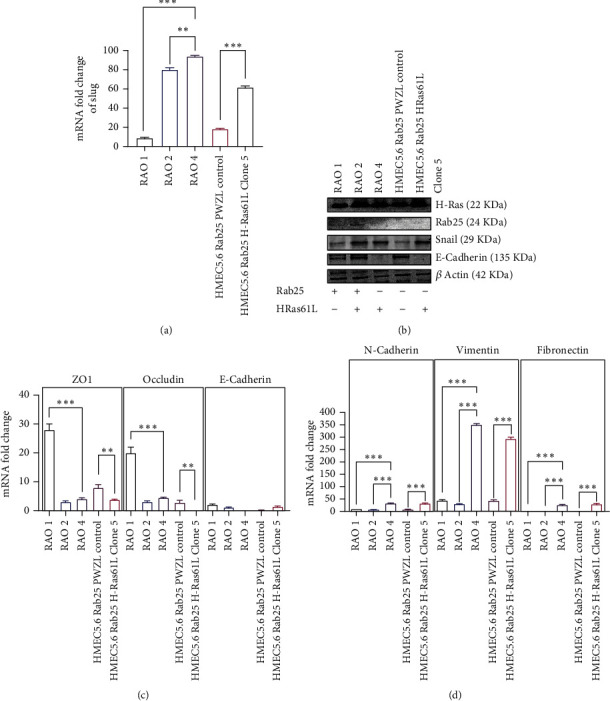
Loss of RAB25 and overexpression of mutant H-Ras61L leads to loss of E-cadherin and drives cells to undergo epithelial to mesenchymal transition (EMT) via transcription factors Snail and Slug. (a) Upregulation of the transcription factor Snail at the protein level in RAO-2, RAO-4, and Clone#5 of HMEC 5.6 Rab25- cells. Loss of E-cadherin expression in HEMC5.6 Rab25- Clone#5 and in positive control RAO-4. (b) Graph showing the upregulation in the mRNA level expression of transcription factor Slug in RAO-2, RAO-4, and Clone#5 of HMEC 5.6 Rab25- (*n* = 3). (c) Graph showing the downregulation in the mRNA level expression of epithelial markers like ZO-1, occludin, and E-cadherin with standard error bars (*n* = 3). (d) Graph showing the upregulation in the mRNA level expression of mesenchymal markers like N-cadherin, vimentin, and fibronectin with standard error bars (*n* = 3). Any starred value indicates a *p* value of less than 0.05.

In addition to standard assays for invasion and migration, the RAO-4 cell line and the HMEC 5.6 Clone#5 line displayed increased mammosphere formation significantly, increasing by at least threefold compared to the nonmalignant lines (Figures 6(a)–6(c)). These nonmalignant lines included RAO-1, which is hTERT immortalized; RAO-2, which is hTERT immortalized and expresses H-RAS61L; and HMEC 5.6 Rab25-, which is a hTERT and CDKR24C immortalized clone that has had RAB25 expression suppressed. Cancer stem cell markers were examined (Figures 6(d) and 6(g)), and CD24 was substantially suppressed in the transformed lines of Rao-4 and HMEC 5.6 Clone#5. CD44 expression was not consistently altered. ALDH1 expression was significantly increased in HMEC 5.6 Clone#5 compared to HMEC 5.6 RAB25- control. This was confirmed by both QRT-PCR and flow cytometry. Claudin1 was lowered very significantly in both transformed cell lines, RAO-4 and HMEC 5.6 Clone#5, suggesting that the combination of the loss of RAB25 and overexpression of H-RAS61L leads to the development of a mesenchymal, claudin-low tumor phenotype. On checking the expression of several EMT factors as outlined in Figure 7, Slug expression was increased at the mRNA level and Snail expression at the protein level (Figures 7(a) and 7(b)). Other factors such as E-cadherin, ZO-1, and occludin also lost expression in the transformed clones of RAO-4 and HMEC 5.6 Clone#5 compared to RAO-1 or HMEC 5.6 RAB25- (Figure 7(c)). Markers such as N-cadherin, vimentin, and fibronectin rose in the transformed clones (Figure 7(d)), implying that the combination of loss of RAB25 and H-RAS61L overexpression was driving immortal HMEC toward a mesenchymal-like, stem cell-enriched tumor. We examined mammary spindle cell carcinomas to corroborate our in vitro findings as they best exemplify the claudin-low, mesenchymal phenotypical breast tumor.

### 3.5. RAB25 Affects HIF-1*α* Protein Levels

Our previous work [17] demonstrated that Rab25 significantly suppressed the levels of VEGF-A, VEGFR-1 mRNA, and VEGFR-1 protein. VEGF regulation may also be influenced by HIF-1 in a hypoxic tumor environment [32–34]. To test if RAB25 mediates via HIF-1*α*, we first conducted a real-time PCR assay to determine if HIF-1*α* levels were affected by RAB25 expression. As shown in Figure 6(e), levels are quite low in RAO-1 and RAO-2 but rise quite significantly in RAO-3 and RAO-4. RAB25 expression brings down HIF-1*α* levels quite significantly in RAO-3 and RAO-4. Protein levels of HIF-1*α* as determined by western blotting mirrored real-time PCR results and are displayed in Figure 6(f).

### 3.6. Cytogenetic Study Shows the Clonal Evolution with Presence of Mutant H-Ras61L and RAB25 Loss

**Figure 8:**
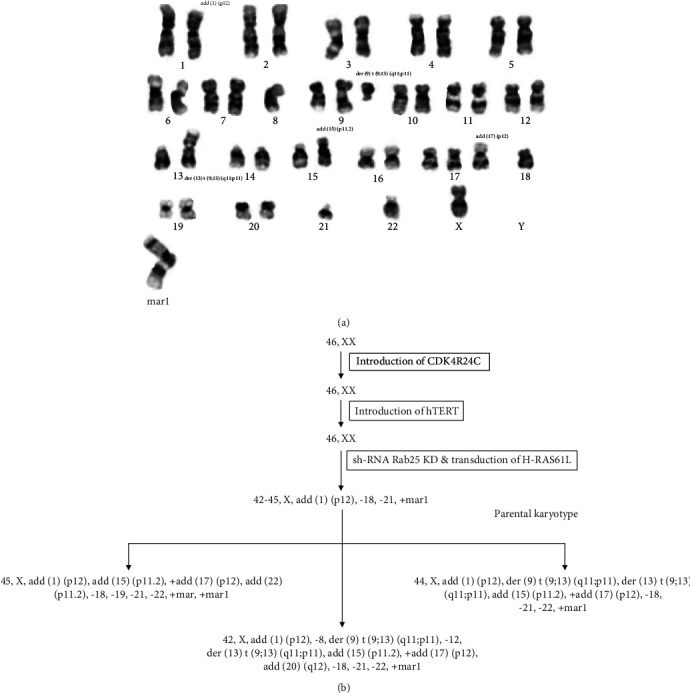
Cytogenetic study shows the clonal evolution with the presence of mutant H-Ras61L and RAB25 loss. (a) A representative G-banded karyotype of HMEC 5.6 Rab25- Clone#5. (b) Summary of the karyotypic changes acquired by HMEC 5.6 Rab25- during immortalization, sh-RNA mediated Rab25KD, and transduction of H-Ras61L and resulting clonal evolution.

Metaphase cells that were analyzed by G-banding showed an abnormal complex karyotype (Figure 8(a)). The sample contained a high proportion of hyperdiploid and hypotetraploid cells (>50%). The remaining cells were hypodiploid (with <46 chromosomes). Only one sex chromosome was identified in the hypodiploid cells. Other clonal chromosomal losses in the hypodiploid cells included -8, -18, -21, and -22. We identified the large marker chromosome (mar1) composed of unknown origin material in all analyzed cells.

The parental karyotype of this cell line was found to be 42-45, X, add (1)(p12), -18, -21, +mar1 (Figure 8(b)).

Clonal structural abnormalities included an abnormal chromosome 1 with additional material of unknown origin on the short arm, band p12 - add (1)(p12) a reciprocal, apparently balanced translocation between the long arm of chromosome 9, band q11, and the short arm of chromosome 13, band p11. This translocation resulted in a derivative chromosome 9 and a derivative chromosome 13 - der(9)t(9;13)(q11;p11),der(13)t(913)(q11;p11), an abnormal chromosome 15 with additional material of unknown origin on the short arm, band p11.2 - add (15)(p11.2), an additional abnormal chromosome 17 with additional material on the short arm, band p12 -+add(17)(p12).

### 3.7. Patient Characteristics and Loss of RAB25 Expression in Clinical Mammary Spindle Cell Tumor Specimens

To understand the significance of the loss of RAB25 expression in spindle cell breast cancer, we expanded our study to look at the expression of RAB25 in clinical samples.

Our series included 13 patients, all females, who ranged in age from 44 to 92 years (median 76 years). Ten patients were Caucasians (76.92%), two were African Americans (15.38%), and ethnicity was unknown in one patient. The tumor size ranged from 1.5 to 7.5 cm (mean 3.2 cm). All cases were clinically of breast origin and showed spindled/sarcomatoid morphology. There were eight cases (61.53%) that exhibited pure spindle morphology of variable appearance, whereas five (38.46%) contained invasive ductal carcinoma comprising <20% of the tumor mass in addition to the spindle cell carcinoma. Ductal carcinoma in situ was present in eight cases (61.53%). One patient (7.69%) exhibited heterologous elements (chondrosarcoma). Fibrocystic changes were present in six patients (46.15%). There were twelve cases (92.3%) with grade 3 and one case (7.69%) with grade 1 differentiation. Two cases (15.38%) showed lymphovascular invasion.

Four patients (30.76%) were staged at IA, six (46.15%) at IIA, one (7.69%) at IIB, one (7.69%) at IIIA, and one (7.69%) at stage IV. Treatment consisted of local excision in four cases (30.76%) or modified radical mastectomy in nine cases (69.23%). In six patients (46.15%) who underwent axillary nodal dissection, lymph node metastases were present in two cases, and one of them had extracapsular extension. Five patients (38.46%) received postoperative chemotherapy. Only one (7.69%) out of thirteen cases had positive ER (treated with letrozole), and another patient (7.69%) had HER2-positive status and received trastuzumab for one year. All patients had negative PR status. One patient who underwent modified radical mastectomy and postop chemotherapy developed lung metastases 4.5 years after the initial diagnosis. One patient had bone metastases at the time of initial diagnosis (who received zoledronic acid). One patient developed invasive ductal carcinoma after two years of initial diagnosis. Median overall survival was 55 months in all patients. Patient details are available in Table 2.

**Figure 9:**
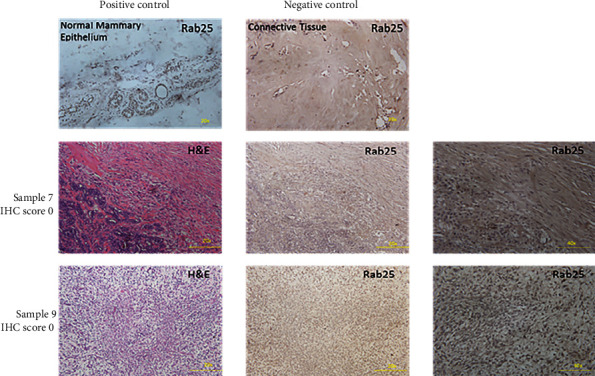
Loss of RAB25 expression correlates with spindle cell carcinoma. Representative images of positive and negative controls and Rab25 in mammary spindle cell carcinoma tumor sections.

A retrospective analysis was done on 13 mammary spindle cell carcinoma samples (Figure 9). We scored the expression of RAB25 in these samples (Table 3). Our results show that 12 of the samples scored 0 or negative for RAB25 expression, and only 1 sample scored 1 or slightly positive for RAB25 expression (Table 4). Although we have used a small group of patients for the study, we obtained a highly significant result for RAB25 loss in spindle cell carcinoma cases. We used normal mammary epithelium tissue for the RAB25 positive control, and the connective tissue served as the negative control. We also looked at the expression level of the transcription factor Snail in spindle cell carcinoma cases. Our results show that five of the samples scored 2 or positive, three samples scored 1 or weak staining, and five samples scored 0 or negative for Snail expression.

### 3.8. H-RAS Mutations Are Strongly Associated with RAB25 Loss and Spindle Cell Carcinoma

We looked at the presence of H-RAS or K-RAS mutations in three of the mammary spindle cell carcinoma cases that were negative for RAB25 expression. We specifically selected these three cases as they exclusively displayed pure spindle cell histology. On RAS mutation study analysis, we see two samples that show the presence of G13V H-RAS-specific mutations, and one of them detected the presence of G13R H-RAS-specific mutation. No K-RAS mutations were detected in our study. Although we have a small set of sequenced samples, the results are significant, with three of the samples carrying H-RAS-specific mutations at the G13 locus (Table 5).

### 3.9. Transformed HMEC with Loss of RAB25 and Overexpression of Mutant H-RAS61L Forms Tumors in Nude Mice

**Figure 10:**
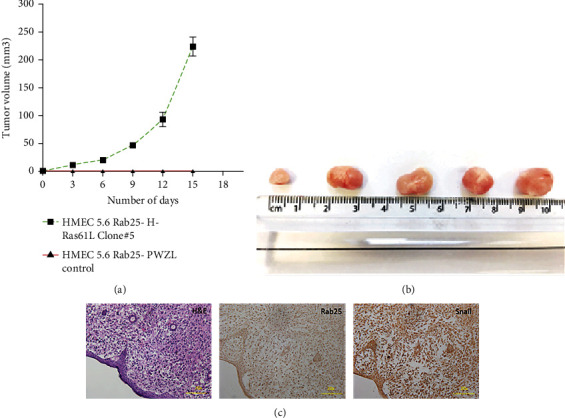
Transformed HMEC with loss of RAB25 and overexpression of mutant H-Ras61L forms tumors in nude mice. (a) Tumor volumes of HMEC 5.6 RAB25- H-Ras61L Clone#5 and HMEC 5.6 RAB25- pWZL control in nude mice (*n* = 5). (b) Representative images of the tumors formed in nude mice injected with HMEC 5.6 RAB25- H-Ras61L Clone#5. (c) Representative images of H&E (20x), Rab25 (negative), and Snail (positive) staining of mice tumor tissue by immunohistochemistry.

A xenograft study in nude mice was carried out to confirm the transformed nature of cells with RAB25 loss and overexpression of H-RAS61L (Figure 10(a)). HMEC 5.6 RAB25- H-RAS61L Clone#5 cells formed tumors in nude mice after one week of injection, with an average tumor size of 220 mm^3^ at the end of 15 days (Figure 10(b)). Tumor formation was not seen in the control group mice, suggesting that loss of RAB25 and overexpression of mutant H-RAS61L are both necessary for transformation. We also looked at the expression of RAB25 and Snail in mouse tumor sections by immunohistochemistry. The xenograft tumor sections were negative for RAB25 expression and showed overexpression of Snail (Figure 10(c)).

## 4. Discussion

Immortalization of cells is a critical step in the transformation of cells [32]. Viral oncogenes like SV40 large T antigen or E6/E7 HPV protein have been extensively used to immortalize cell lines and study them. In our study, we immortalized cell lines with a set of defined genetic elements in an attempt to better understand the role of oncogenes and tumor suppressors. Human mammary epithelial cells (HMECs) were obtained from a healthy individual undergoing reduction mammoplasty at the SIU School of Medicine. The primary epithelial cell lines were immortalized by transducing them with LXSN CDK4 R24C—a mutant form that cannot be inhibited by p16 INK4A—followed by transduction with hTERT, the catalytic subunit of the telomerase enzyme.

Our results show that the cooccurrence of the loss of RAB25 and the expression of mutant H-RAS61L is necessary for cells to transform. As seen in HMEC 5.6 RAB25- Clone#5 cells, the effects of this genetic cooccurrence were demonstrated by functional in vitro assays confirming increased migration, invasion, and colony-forming abilities. RAO-2 cells that express mutant H-RAS and RAB25 and other RAB25-negative clones with no RAS overexpression do not show any significant changes in any in vitro assays that confirm transformation, proving that the expression of the oncogene H-RAS61L alone is insufficient and RAB25 loss is required for cells to transform. We also noticed that the ability of cells to undergo transformation is dependent on the gene dosage of the oncogene. As the level of overexpression of the oncogene increased, cells had a higher tendency to undergo transformation. Our findings show that transformed HMEC with loss of RAB25 and mutant H-RAS61L have very low levels of CD24 and Claudin1, while the level of CD44 remains significantly high.

Additionally, the formation of primary and secondary mammospheres confirmed the stem-like nature of these cells. Several studies confirm that claudin-low tumors are enriched with stem-like properties [33, 34]. HMEC 5.6 RAB25- Clone#5 cells show overexpression of transcription factors Snail and Slug, leading to E-cadherin loss, one of the characteristic features of EMT. Overexpression of Snail could be a potential inducer of EMT by repressing E-cadherin's expression [35].

**Figure 11:**
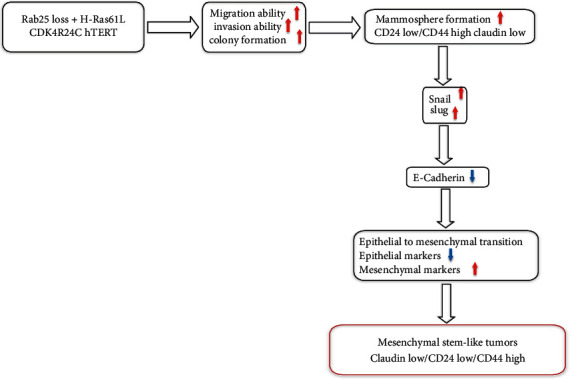
Stepwise progression of transformation of HMEC 5.6 Rab25- cells giving rise to mesenchymal stem-like tumors.

In this study, we show that with RAB25 loss and the presence of mutant H-Ras61L, cells lose E-cadherin expression and undergo epithelial-to-mesenchymal transition, a critical process in cancer progression and metastasis. This result is supported by the downregulation of epithelial marker expressions such as E-cadherin, occludin, and ZO-1 and the upregulation of mesenchymal markers like N-cadherin, vimentin, and fibronectin. Our transformed cell lines overexpressed the transcription factors Snail and Slug, which are involved in epithelial-to-mesenchymal transition [35–37]. Both RAB25 loss and overexpression of mutant oncogene appear to be necessary for cells to lose E-cadherin expression and undergo EMT. Likewise, the transformed cell lines, RAO-3 and RAO-4, displayed enhanced levels of HIF-1 alpha, which were brought down with Rab25 expression. Cancer stem cells can be critically dependent on the HIF-1 alpha pathway [38], and the effects of Rab25 may in part be controlling the stemness of the cells. Recent studies have highlighted the connection between Snail and cancer stem cells [39, 40] and that Snail-driven cancer cells that undergo EMT acquire stem-like traits and express CD24low/CD44high levels [41]. We have proposed a stepwise model in Figure 11.

The results obtained from our study so far reveal that HMEC 5.6 RAB25- Clone#5 and RAO-4 cells can give rise to the claudin-low subtype of triple-negative breast cancers. This subtype of triple-negative breast cancer is molecularly characterized by a stem-like nature with CD44high/CD24low levels and undergoes EMT [31], and this is consistent with our findings.

Genetic abnormalities are common in different cancers. In our study, cells likely first acquired the karyotype of 42-45, X, add (1) (p12), -18, -21, +mar1. A fraction of the cells underwent further chromosomal rearrangements to give rise to three related cell populations, having additional chromosomal abnormalities including der(9)t(9;13)(q11;p11),-12,der(13)t(9;13)(q11;p11), add(15)(p11.2),+add(17)(p12), add(22)(p11.2), and -22. Several genes found on chromosome 18, such as SMAD4, SMAD2, MIB1, and MBD1, are involved in breast cancer development and progression. Chromosomal abnormalities of chromosome 1p/q are highly associated with multiple myeloma conditions [42]. TRPM2, ITGB2, and ETS2 are located on chromosome 21 and are associated with breast, ovarian, and prostate cancers. Deletion of a region of chromosome 22 is common in breast cancer and also in colorectal cancers [43, 44]. It could lead to the loss of function of tumor suppressor genes at this location and in the nearby regions on chromosome 22 that could play a role in tumor development. The possibility of tumor suppressor genes on the X chromosome was raised a few years ago, and studies suggested that the loci on the X chromosome carry tumor suppressor genes [45]. Loss of X chromosome may cause complete loss of function of tumor suppressor genes and result in individuals becoming more susceptible to cancer formation [46, 47]. Additionally, the analyzed cells also carried a large marker chromosome composed of material of unknown origin.

Our study indicates that spindle cell carcinoma could present at an early or late age. They have variable morphology, a strong association with invasive ductal carcinoma (38.46%) and ductal carcinoma in situ (61.53%), and are poorly differentiated (92.3%). The majority of these breast cancers are triple-negative (84.61%). Most patients are treated by lumpectomy and/or mastectomy with axillary node evaluation, often combined with postoperative chemotherapy. Spindle cell carcinoma has the potential for both local recurrence and distant metastasis. Our study shows that Rab25 loss is strongly associated with spindle cell carcinoma, and 92% of our tumor samples were negative for RAB25 expression. Loss of RAB25 in breast cancer is not noted in CBioPortal data, but our previous studies indicate that it can occur with significant frequency in triple-negative breast cancers [15, 17].

Likewise, the H-Ras mutation is found in 0.3% of breast cancer cases in CBioPortal data. Mammary spindle carcinoma may represent a distinct clinicopathologic entity that is characterized by, among other things, loss of RAB25 and activation mutations of p21 RAS.

Mutations in RAS genes have been associated with the development and progression of various cancers [48, 49]. Our RAS mutational study detected the presence of H-RAS-specific mutation at the G13 loci consistently in three pure mammary spindle cell carcinoma cases. These three tumors did not express RAB25. N-RASQ61R and N-RASG12D mutations show functional similarity by activating PI3K and MAPK pathways in melanoma cases [50]. We have already shown the transforming ability of the H-RASQ61L mutant in the absence of RAB25 in our in vitro studies, and we think that H-RASG12D may likewise have a similar functional ability to the H-RASQ61L mutant. Additional studies with a larger sample size to screen for H-RAS-specific mutations can confirm our findings.

The exact mechanism of action governing the cooperation between RAB25 loss and H-RAS61L that contributes to HMEC transformation remains unknown. The 61L mutation of RAS requires the binding of RAF to stabilize and maintain the oncogenic nature of the protein [51], and it may be that RAF binds to RAB25 under normal circumstances just as it binds to Rap [52]. The loss of RAB25 may free RAF to bind RAS 61L and enhance the oncogenic activity of RAS61L. Clinically, these RAB25-negative tumors may be vulnerable to therapeutic strategies involving synthetic lethality as they have lost RAB25 and may be sensitive to agents that target vesicular trafficking. Historically, triple-negative breast cancers have lacked a therapeutic target, and targeting the ras oncogene has been challenging, but the recent FDA approval of sacituzumab govitecan-hziy has shown that therapeutic targeting is possible as over 90% of these tumors express the Trop-2 protein [53]. Likewise, antibody-drug conjugates that can target these tumors and deliver a synthetically lethal payload to exploit the loss of the rab25 pathway may be an effective therapeutic strategy to target this subset of ras mutant triple-negative breast cancers.

## Figures and Tables

**Table 1:** Primers used to determine mRNA expression of different genes.

Gene	Primer 5′→3′
B-Actin forward	TGGGTCAGAAGGATTCCTATGT
B-Actin reverse	CAGCCTGGATAGCAACGTACA
RAB25 forward	TCGTGGGTAACAAAAGTGACC
RAB25 reverse	AGCTCAACATTGGTAGAGTCC
H-RAS forward	GGATCCATGACGGAATATAAG
H-RAS reverse	TGTGTGCTCTCCTGAGAATTC
CD24 forward	CTCCTACCCACGCAGATTTATTC
CD24 reverse	AGAGTGAGACCACGAAGAGAC
CD44 forward	CTGCCGCTTTGCAGGTGTA
CD44 reverse	CATTGTGGGCAAGGTGCTATT
ALDH1 forward	CCGTGGCGTACTATGGATGC
ALDH1 reverse	GCAGCAGACGATCTCTTTCGAT
CLDN1 forward	CCTCCTGGGAGTGATAGCAAT
CLDN1 reverse	GGCAACTAAAATAGCCAGACCT
Slug forward	TGTTGCAGTGAGGGCAAGAA
Slug reverse	GACCCTGGTTGCTTCAAGGA
E-Cadherin forward	CGAGAGCTACACGTTCACGG
E-Cadherin reverse	GGGTGTCGAGGGAAAAATAGG
Occludin forward	CCCCATCTGACTATGTGGAAAGA
Occludin reverse	AAAACCGCTTGTCATTCACTTTG
ZO-1 forward	CAACATACAGTGACGCTTCACA
ZO-1 reverse	CACTATTGACGTTTCCCCACTC
N-Cadherin forward	GGTGGAGGAGAAGAAGACCAG
N-Cadherin reverse	GGCATCAGGCTCCACAGT
Vimentin forward	TCTACGAGGAGGAGATGCGG
Vimentin reverse	GGTCAAGACGTGCCAGAGAC
Fibronectin forward	CCACCGTCTCAACATGCTTAG
Fibronectin reverse	CTCGGCTTCCTCCATAACAAGTAC
hTERT forward	AATCCGTCGAGCAGAGTT
hTERT reverse	GCGCGGCTTACCCTTACCCTTACCCT

**Table 2:** Tabulation of the clinical parameters of the 13 patient samples.

Patient ID	Age	Race	Size (cm)	Stage	DCIS	Grade	LVI	LN	ER/PR/HER2	RAB25 status	Fibrocystic changes
Patient 1	57	C	1.8	IA	DCIS+	3	NA	20-	ER/PR/HER2-	Negative	N
Patient 2	78	AA	3.5	IIA	DCIS-	3	No LVI	1-	ER/PR/HER2-	Negative	N
Patient 3	92	AA	7.5	IV	DCIS+	3	LVI+	Nx	ER+ but PR/HER2-	Negative	N
Patient 4	64	C	2.7	IIA	DCIS-	3	No LVI	1-	ER/PR/HER2-	Negative	Y
Patient 5	76	NA	2.1	IIA	NA	3	LVI+	1-	ER/PR/HER2-	Negative	N
Patient 6	82	C	3	IIA	NA	3	NA	6-	ER/PR/HER2-	Negative	N
Patient 7	69	C	1.5	IA	DCIS	3	NA	3-	ER/PR-	Negative	N
Patient 8	44	C	3	IIA	DCIS+	3	1/20LN+ with no LVI extracapsular extension		ER/PR- and HER2+	Positive	Y
Patient 9	76	C	1.9	IA	DCIS+	3	No LVI	2-	ER/PR/HER2-	Negative	N
Patient 10	90	C	6	IIB	DCIS+	3	No LVI	7-	ER/PR-	Negative	Y
Patient 11	73	C	5.5	IIIA	DCIS+	3	NA	3+/13	ER/PR/HER2-	Negative	Y
Patient 12	51	C	2.2	IIA	DCIS-	3	No LVI	5-	ER/PR/HER2-	Negative	Y
Patient 13	88	C	1.5	IA	DCIS+	1	No LVI	2-	ER/PR-	Negative	Y

**Table 3:** Scale used to score IHC sections for Rab25 expression.

IHC score	0	1	2
Intensity of staining	Negative/no staining	Weak	Strong

Fisher's exact test *P* value: 0.0071.

**Table 4:** Tabulation of the total number of patient samples (Fisher's exact test, *n* = 13) scored for Rab25 expression.

Rab25 profile	RAB25+	RAB25-
Number of samples	1	12

**Table 5:** Tabulation of the three samples that were sequenced to detect to presence of H-Ras and K-Ras specific point mutations.

Sample ID	H-Ras mutations	K-Ras mutations
Sample 1	G13V	None
Sample 2	G13R	None
Sample 3	G13V	None

## Data Availability

Data for our study was generated in our laboratory and has been stored locally. Any reasonable request for data will be considered by the corresponding author.
